# Epidemiological characteristics and risk factors of T2DM in Chinese premenopausal and postmenopausal women

**DOI:** 10.1186/s12944-019-1091-7

**Published:** 2019-07-17

**Authors:** Qingjun Li, Xiaoqing Wang, Yaojun Ni, Hairong Hao, Ziyu Liu, Surong Wen, Xiaojuan Shao, Xiaojuan Wu, Weinan Yu, Wen Hu

**Affiliations:** 1 Department of Endocrinology and Metabolism, Huai’an Hospital Affiliated to Xuzhou Medical College and Huai’an Second People’s Hospital, Huai’an, 223001 China; 2 Department of Cardiothoracic Surgery, Hospital Affiliated to Nanjing Medical College and Huai’an First People’s Hospital, Huai’an, 223001 China

**Keywords:** Menopausal, T2DM, Prevalence, Risk factors

## Abstract

**Objective:**

This study was to analyse the prevalence of type 2 diabetes mellitus (T2DM) in premenopausal and postmenopausal women.

**Methods:**

A total of 3227 women met the requirements from June to December in 2014, including 207 cases of premenopausal women and 3020 cases of postmenopausal women. The prevalence of T2DM and the associated risk factors in the two groups were analysed.

**Results:**

The prevalence of premenopausal women with T2DM was 12.1%, while the prevalence in postmenopausal women was 19.4% (*P* < 0.05). Total serum protein (TP) (OR = 1.164 95% CI = 1.023–1.324) (*P* = 0.021) is a major risk factor for premenopausal women with T2DM. The prevalence of T2DM increased with the increase in TP. In postmenopausal groups, the prevalence of T2DM was associated with age (OR = 1.037 95% CI = 1.024–1.051) (*P* < 0.001), BMI (OR = 1.076 95% CI = 1.044–1.109) (P < 0.001), blood pressure (OR = 1.521 95% CI = 1.234–1.875) (*P* < 0.001), triglycerides (TG) (OR = 1.106 95% CI = 1.027–1.190) (*P* = 0.008), blood urea nitrogen (BUN) (OR = 1.065 95% CI = 1.004–1.129) (*P* = 0.036), alanine aminotransferase (ALT) (OR = 1.009 95% CI = 1.003–1.016) (*P* = 0.004) and TP (OR = 1.031 95% CI = 1.005–1.057) (*P* = 0.018).

**Conclusions:**

Postmenopausal women have a higher rate of type 2 diabetes than premenopausal women. TP is a major risk factor for premenopausal women with T2DM. TP, ALT, and BUN are postmenopausal risk factors in addition to traditional risk factors such as obesity, lipidaemia and blood pressure. We should monitor risk factors and take early prevention and intervention measures to reduce the prevalence of diabetes and improve the quality of life of postmenopausal women.

**Trial registration:**

ChiCTR, ChiCTR-TRC-14005029. Registered 29 July 2014,http://www.chictr.org.cn/showproj.aspx?proj=4545

## Introduction

According to epidemiological surveys, the prevalence of diabetes mellitus has gradually increased, and type 2 diabetes (T2DM) accounts for more than 90% of the total number of diabetic patients [1]. The risk of developing T2DM is increased in postmenopausal women [2], and T2DM increases the risk of early menopause [3]. T2DM, the most common chronic disease in postmenopausal women, is a major cause of cardiovascular disease and a leading cause of death among women in Western societies [4]. The pathogenesis of T2DM is complex, and the role of traditional risk factors does not completely explain its physiological and pathological processes. Ageing, obesity and other epidemiological factors play a role in diabetes, and Asians are susceptible to diabetes according to the Chinese T2DM prevention guidelines [1]. In 2013, Heianza Y et al. retrospectively analysed the data of 29,189 males, 6308 premenopausal females and 4570 postmenopausal females in Japan. They found that postmenopausal status was significantly associated with abnormal blood glucose. Women at the age of menopause or older may have abnormally elevated blood glucose levels [5]. At present, studies have suggested that endogenous levels of sex hormones may play important roles in the pathogenesis of T2DM [6]. However, there is still insufficient data on the prevalence and risk factors for T2DM in Asian premenopausal and postmenopausal women.

However, there are few systematic analyses on the risk factors of T2DM before and after menopause at home and abroad.

Total serum protein (TP) is the sum of all the proteins in the serum, most of which are synthesized by the liver. TP functions lie in nutrition, the buffer and maintenance of colloid osmotic pressure, delivery, immunity, coagulation, fibrinolysis and various enzyme reactions [7]. Moreover, TP plays an important role in understanding the nutritional status of the body as well as liver and kidney disease diagnosis, treatment and monitoring [8]. A study concluded that serum TP level varied in non-diabetes and diabetes [9].However,it is not clear whether there is a correlation between serum TP level and the risk of T2DM.

This study explored the prevalence of T2DM in Chinese premenopausal and postmenopausal women and evaluated both traditional and non-traditional risk factors for T2DM. It can provide a reference for effective prevention and treatment of T2DM.

## Methods

### Ethics statement

This cross-sectional study was part of the Huaian Diabetes Prevention Program (ChiCTR-TRC-14005029) and was approved by the Huaian Second Hospital Ethics Committee, XuZhou Medical University, China. Written informed consent was obtained from all participants in this study.

Study population: In the present study, 4246 subjects (aged 40–79 years, females with regular menstruation or menopausal for more than 1 year) with annual routine health examinations between August and December 2014 at the health examination centre of Huaian Second Hospital, Affiliated Hospital of Xuzhou Medical College in Huaian (Jiangsu, China), were enrolled. Women with regular menstrual cycles were defined as the premenopausal group. Postmenopausal was defined as at least 12 months without a menstrual period (exclusion of drugs, pregnancy, severe weight loss and other pathological reasons for cessation of menses) [10]. Subjects were subsequently excluded from the analysis if any of the following criteria applied:

#### Exclusion criteria

1) Thyroid and adrenal related diseases (*n* = 63); 2) Serious liver and kidney disease (*n* = 67); 3) Myocardial infarction, acute cerebrovascular accident (*n* = 21); 4) Microalbuminuria (mALB) > 20 mg/L (*n* = 117); 5) Incomplete information (*n* = 109); 5) Type 1 diabetes, impaired fasting glucose and impaired glucose tolerance (*n* = 606); and 6) Surgery, chemotherapy or radiation-induced premature menopause and hormone replacement therapy (*n* = 36). Therefore, 3227 women (207 premenopausal, 3020 postmenopausal) were deemed eligible for the analysis.

### Data collection

Well-trained research investigators conducted a structured questionnaire, measured body height, weight and waist circumference (WC) to calculate body mass index (BMI), and measured blood pressure and pulse rate. The maximum blood pressure after 3 measurements was recorded; the pulse rate was measured continuously, and three readings were averaged. (OMRON Model HEM-752 FUZZY, Omron Company, Dalian city, Liaoning Province, China). The questionnaire collected information about past medical history, medication history, history of smoking and drinking, childbearing history, and history of sleep disorders (excessive daytime somnolence, insomnia, abnormal movements or behaviours during sleep, and an inability to sleep at the desired time) [11].

Fasting venous blood samples were collected for determination of fasting plasma glucose (FPG) and related biochemical parameters, including total cholesterol (TC), triglycerides (TG), high-density lipoprotein cholesterol (HDL-C), low-density lipoprotein cholesterol (LDL-C), creatinine (CREA), uric acid (UA), blood urea nitrogen (BUN), alanine aminotransferase (ALT), aspartate transaminase (AST), TP, and total bilirubin (TBIL) (VARIANT II and D-10 Systems, BIO-RAD, Hercules, CA, USA).

Glycated haemoglobin (HbA1c) was measured by high performance liquid chromatography (Variant II and D-10 Systems, Bio-Rad Laboratories Inc., Hercules, CA, USA). The labile glycated fraction was removed by incubation of washed,packed erythrocytes in saline at 37 °C for at least 5 h, with subsequent lysis of the erythrocytes in water. Cellular debris was removed by mixing with CCl4 followed by centrifugation; the hemolysate sample was injected on the HPLC column. The HbA1c fraction eluted after HbA1a and HbA1b and before the HbA0 fractions. Percentage HbA1c was calculated by dividing the area under the HbA1c peak by the total area under all peaks. All quality control criteria of the ADA were applied for HbA1c measurement [12], and the laboratory was properly qualified. The estimated glomerular filtration rate (eGFR) was calculated from creatinine levels using the CKD-EPI formula [13]. People without a prior diabetes history underwent glucose tolerance testing (morning fasting, oral 82.5 g with one molecule of water glucose powder), and people with a past history of diabetes underwent a bread glycaemic test (including 100 g flour). Blood sugar (2 h PG) was measured 2 h after taking the sugar or 2 h after the meal. Diabetes was defined according to the 2012 ADA criteria [7]: FPG ≥126 mg/dl (7.0 mmol/L); or a 2 h plasma glucose in the 75-g oral glucose tolerance test (OGTT) ≥200 mg/dL (11.1 mmol/L); and/or HbA1c ≥6.5%.

### Statistical analysis

All statistical analyses were performed using SPSS version 19.0 (SPSS Inc., Chicago, IL). Each variable was tested for normal distribution using the Kolmogorov-Smirnov test. Non-normally distributed variables were analysed with a Mann-Whitney U-test for two groups. Data are presented as a percentage, median (25th and 75th percentiles) or mean ± standard deviation (SD). BMI, WC, SBP, DBP, FPG, HbA1C, TG, TC, ALT, AST, LDL-C, HDL-C, CREA, eGFR, BUN and TP were log-transformed due to their non-normal distribution for correlation. We compared the clinical and metabolic indexes between the diabetic and non-diabetic groups before and after menopause. Student’s t-test was used for comparing normally distributed variables, the Mann-Whitney U-test was used for comparing non-normally distributed variables, and the Chi-square test was used for comparisons of categorical variables. The multivariate logistic regression analysis was performed to determine the independent variables associated with premenopausal and postmenopausal women with T2DM. To better investigate the relationship between TP and T2DM, we constructed unadjusted and multivariable-adjusted models (incorporating age and WC, hypertension, ALT, BUN, and GFR). A value of *P* < 0.05 was considered statistically significant.

## Results

### Prevalence of T2DM and characteristics of participants in the premenopausal and postmenopausal groups

The total population was divided into two groups (premenopausal and postmenopausal). As shown in Table 1, the percentage of T2DM was 18.9% in 3227 women, of which 12.1% were premenopausal and 19.4% were postmenopausal. The prevalence of T2DM in postmenopausal women was significantly higher than in premenopausal women (*P* < 0.01). In addition, nearly all of the characteristics evaluated differed between the two groups, with the exception of BMI, WC, hypertension, drinking, history of CHD, HDL-C, and TBIL.

**Table 1:** Comparison of related indexes before and after menopause in the general population

	Total population		Z/χ^2^	*P*
	Premenopausal (*n* = 207)	Postmenopausal (*n* = 3020)		
Age (years)	46.65 ± 1.78	60.61 ± 7.55	−75.59	< 0.001
BMI (kg/m^2^)	24.01 (22.19, 25.86)	24.46 (22.43, 26.67)	−1.94	0.05
WC (cm)	78 (73, 81)	81 (78, 88)	−7.43	< 0.001
SBP (mmHg)	128 (120, 138)	135 (124, 150)	−6.31	< 0.001
DBP (mmHg)	79 (71, 86)	82 (75, 90)	−3.66	< 0.001
HbA1c (%)	5.4 (5.1, 5.8)	5.7 (5.4, 6)	−6.25	< 0.001
FPG (mmol/L)	5.19 (4.89, 5.53)	5.46 (5.11, 5.98)	−6.62	< 0.001
TC (mmol/L)	4.72 (4.21, 5.38)	5.24 (4.67, 5.82)	−7.60	< 0.001
TG (mmol/L)	1.48 (1.12, 2.01)	1.77 (1.39, 2.35)	−6.04	< 0.001
LDL-C (mmol/L)	2.44 (2.07, 2.76)	2.74 (2.3, 3.2)	−6.53	< 0.001
HDL-C (mmol/L)	1.4 (1.21, 1.6)	1.37 (1.18, 1.59)	−0.71	0.48
CREA (μmol/L)	57.7 (53.4, 64.3)	61.5 (54.9, 68.8)	−4.14	< 0.001
UA (μmol/L)	234.2 (201.7, 267.1)	252.9 (214.13, 298.78)	−4.82	< 0.001
BUN (mg/dl)	4.43 (3.74, 5.29)	4.95 (4.24, 5.8)	−5.81	< 0.001
ALT (U/L)	18 (14, 25)	20 (16, 26)	−4.27	< 0.001
AST (U/L)	18 (16, 22)	20 (17, 24)	−5.47	< 0.001
TP (g/L)	73 (70.7, 75.3)	74.1 (71.7, 76.5)	−3.93	< 0.001
TBIL (μmol/L)	9.97 (7.85, 13.57)	10.875 (8.67, 13.78)	−2.25	0.03
eGFR (ml/min)	105.7261 (98.49, 109.27)	92.9344 (81.88, 99.67)	−14.26	< 0.001
T2DM n (%)	25 (12.1)	586 (19.4)	6.775	0.008
Smoking n (%)	4 (1.9)	42 (1.4)	0.40	0.53
Drinking n (%)	6 (2.9)	34 (1.1)	4.97	0.03
History of Hypertension n (%)	25 (12.1)	1044 (34.6)	44.24	< 0.001
History of CHD n (%)	6 (2.9)	169 (5.6)	2.75	0.97
History of sleep disorders n (%)	3 (1.4)	99 (3.3)	2.12	0.15

Data are presented as the mean ± SD, number (as %), or median (range), as appropriate. Comparisons between the three groups were made using ANOVA or Fisher’s exact test, as appropriate. International system of units (SI) conversion: *BMI* body mass index, *CHD* Coronary heart disease, *SBP* systolic blood pressure, *DBP* diastolic blood pressure, *TC* total cholesterol, *TG* triglycerides, *UA* uric acid, *BUN* blood urea nitrogen, *CREA* serum creatinine, *e-GFR* estimated glomerular filtration rate, *FPG* fasting plasma glucose, *HDL-c* high-density lipoprotein cholesterol, *LDL-c* low-density lipoprotein cholesterol, *TP* Total Protein

### Comparison of the clinical and metabolic indexes in the diabetic and non-diabetic groups before and after menopause

As shown in Table 2, the 207 premenopausal females were divided into two groups according to diabetes definition (25 diabetics with a mean age of 46.65 ± 1.38 years, 182 non-diabetics with a mean age of 46.68 ± 1.83 years). History of hypertension, WC, FPG, HbA1c, and TP in diabetic group were significantly higher than in the non-diabetic group (*P* < 0.05). Moreover, the two groups included 586 diabetics with a mean age of 60.05 ± 7.39 years and 2434 non-diabetics with a mean age of 62.93 ± 7.75 years from a total of 3020 postmenopausal females. Many factors, including age, BMI, WC, history of hypertension, history of CHD, SBP, DBP, HbA1c, FPG, TG, ALT, TP, BUN and UA, in the diabetic group were significantly higher than in the non-diabetic group (*P* < 0.05).

**Table 2:** Baseline comparison of relevant indexes before and after menopause

	Premenopausal		*t/Z/χ* ^*2*^	*P*	Postmenopausal		*t/Z/χ* ^*2*^	*P*
	Non-diabetic (*n* = 182)	Diabetic (*n* = 25)			Non-diabetic (*n* = 586)	Diabetic (*n* = 2434)		
Age (years)	46.65 ± 1.83	46.68 ± 1.38	0.083	0.934	60.05 ± 7.39	62.93 ± 7.75	8.158	< 0.001
BMI (kg/m^2^)	23.83 (21.975, 25.79)	24.91 (23.05, 27.78)	−1.896	0.058	24.27 (22.22,26.31)	25.39 (23.03, 27.99)	−7.181	< 0.001
WC (cm)	78 (73, 81)	81 (78, 90.5)	−2.553	0.011	81 (77, 84.25)	84 (80, 91)	−10.08	< 0.001
SBP (mmHg)	127.5 (120, 136.25)	130 (119.5, 162.5)	−1.324	0.185	134 (123, 149)	140 (130, 158.25)	−7.422	< 0.001
DBP (mmHg)	79 (70, 85)	85 (72.5, 92)	−1.616	0.106	81 (74, 90)	84 (76, 93.25)	−3.258	0.001
HbA1c (%)	5.4 (5.1, 5.7)	6.4 (5.6, 8.7)	−5.001	< 0.001	5.6 (5.3, 5.9)	6.8 (6.1, 7.8)	−29.113	< 0.001
FPG (mmol/L)	5.18 (4.88, 5.48)	5.43 (5.06, 8.52)	−2.949	0.003	5.35 (5.06, 5.74)	6.975 (5.67, 8.53)	−23.812	< 0.001
TC (mmol/L)	4.735 (4.21, 5.36)	4.51 (4.15, 5.6)	−0.429	0.668	5.24 (4.68, 5.8)	5.2 (4.63, 5.88)	−0.014	0.989
TG (mmol/L)	1.465 (1.12, 1.99)	1.55 (1.17, 2.17)	−0.815	0.415	1.73 (1.37, 2.28)	1.92 (1.5, 2.61)	−5.974	< 0.001
LDL-C (mmol/L)	2.445 (2.09, 2.76)	2.32 (1.915, 3.14)	−0.249	0.803	2.73 (2.31, 3.2)	2.75 (2.28, 3.2)	−0.010	0.992
UA (μmol/L)	236.8 (203.18,267.13)	227.8 (197.4, 269.7)	−0.274	0.784	251.9 (213.3294.5)	261.05 (218.5324.3)	−3.537	< 0.001
BUN (mg/dl)	4.405 (3.73, 5.22)	4.91 (3.75, 6.34)	−1.472	0.141	4.93 (4.21, 5.77)	5.055 (4.37, 6.01)	−2.808	0.005
ALT (U/L)	18 (14, 25)	19 (14, 25)	−0.483	0.629	20 (16, 25)	22 (18, 29)	−6.021	< 0.001
AST (U/L)	18 (15.75, 23)	16 (15.5, 21.5)	−0.662	0.508	20 (18, 24)	20 (17, 24)	−0.491	0.623
TP (g/L)	72.8 (70.6, 75)	75 (73.15, 77.55)	−3.084	0.002	74 (71.6, 76.3)	74.4 (72.2, 77)	−3.131	0.002
TBIL (μmol/L)	9.995 (7.84, 13.59)	9.15 (7.81, 13.56)	−0.564	0.572	10.845 (8.66,13.69)	10.99 (8.68, 14.03)	−0.620	0.535
eGFR (ml/min)	105.48 (97.45,108.81)	108.13 (98.56,111.67)	−1.718	0.086	92.93 (82.12,99.73)	92.66 (79.75, 99.41)	−1.068	0.286
Smoking n (%)	4 (2.2)	0 (0)	0.560	0.454	34 (1.4)	8 (1.4)	0.003	0.953
Drinking n (%)	4 (2.2)	2 (8)	2.630	0.105	32 (1.3)	2 (0.3)	4.020	0.045
History of Hypertension n (%)	17 (9.3)	8 (32)	10.630	0.001	751 (20.9)	293 (50)	76.536	< 0.001
History of CHD n (%)	4 (2.2)	2 (8)	2.630	0.110	115 (4.7)	54 (9.2)	18.030	< 0.001
History of sleep disorders n (%)	3 (1.6)	0 (0)	0.420	0.520	68 (2.8)	31 (5.3)	9.283	0.002

Data are presented as the mean ± SD, number (as %), or median (range), as appropriate. Comparisons between the three groups were made using ANOVA or Fisher’s exact test, as appropriate International system of units (SI) conversion: *BMI* body mass index, *CHD* Coronary heart disease, *SBP* systolic blood pressure, *DBP* diastolic blood pressure, *TC* total cholesterol, *TG* triglycerides, *UA* uric acid, *BUN* blood urea nitrogen, *e-GFR* estimated glomerular filtration rate, *FPG* fasting plasma glucose, *LDL-C* low-density lipoprotein cholesterol, *TBIL* Total bilirubin, *TP* Total Protein

### Multivariate logistic regression analysis for T2DM in premenopausal and postmenopausal females

**Fig. 1:**
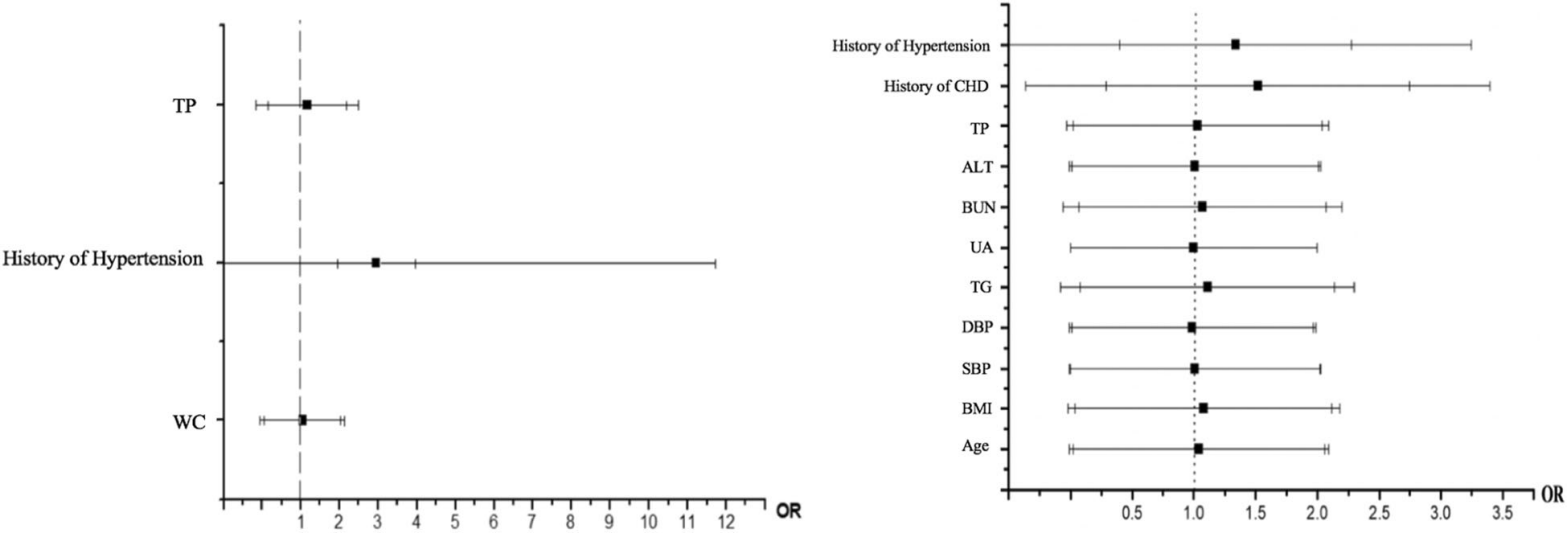
Multiple logistic regression analyses of the odds ratio for T2DM in the premenopausal and postmenopausal groups. BMI body mass index; WC waist circumference; SBP systolic blood pressure; DBP diastolic blood pressure; TG triglycerides; BUN blood urea nitrogen; TG triglycerides; UA uric acid; BUN blood urea nitrogen; TP total protein

Multivariate logistic regression analysis was used to investigate the risk factors for T2DM in premenopausal and postmenopausal groups, as shown in Fig. 1. In the premenopausal group, with T2DM as the dependent variable, WC, TP, and hypertension history were independent variables for multivariate logistic regression analysis. The results showed that TP was an independent risk factor of T2DM. In the postmenopausal group, with BMI, WC, SBP, DBP, TG, TP, UA, ALT, BUN, history of hypertension, and history of CHD as independent variables, multivariate logistic regression analysis was performed. The results showed that history of hypertension, SBP, DBP, TG, ALT, TP and BUN were risk factors for T2DM in postmenopausal women (*P* < 0.05).

### Multivariate logistic regression analysis of the relationship between TP and T2DM in the total population

TP was an independent risk factor for T2DM in the premenopausal and postmenopausal groups. To better investigate the relationship between TP and T2DM, the subjects were divided into four groups based on stratification of TP levels using the 25th, 50th and 75th percentiles as cut-off points. The groups were Q1, TP < 71.6 g/L; Q2, TP 71.7–74.0 g/L; Q3, TP 74.1–76.3 g/L; and Q4, TP > 76.4 g/L. As shown in Table 3, Model 1 was adjusted for age and WC, subjects in the highest quartile of serum TP were more likely to have T2DM (OR = 1.46, 95% CI = 1.13–1.89) (*P* = 0.004). After adjustment for TC, TG, LDL-C and HDL-C + model 1 (model 2).subjects in the highest quartile of serum TP still had higher odds of having T2DM (OR = 1.41, 95% CI = 1.09–1.83) (*P* = 0.010). After adjustment for SBP, DBP, ALT, AST, BUN, TBIL, and eGFR + model 2 (model 3), the correlation remained. Compared to subjects in the lowest quartile of TP, those in the highest quartile had higher odds of having T2DM (OR = 1.42 95% CI = 1.09–1.85) (*P* = 0.011).

**Table 3:** Relationship between the prevalence of T2DM and TP

TP(g/L)	Q1, *n* = 810	Q2, *n* = 816		Q3, *n* = 786		Q4, *n* = 815	
	69.9 (59.3–71.6)	73.0 (71.7–74.0)		75.1 (74.1–76.3)		78.2 (76.4–90.9)	
Models							
T2DM	Reference	*OR (95% CI)*	*P*-value	*OR (95% CI)*	*P*-value	*OR (95% CI)*	*P*-value
Model1	1	1.19 (0.92 1.56)	0.186	1.24 (0.95 1.61)	0.116	1.46 (1.13 1.89)	0.004
Model2	1	1.19 (0.91 1.56)	0.203	1.22 (0.95 1.60)	0.142	1.41 (1.09 1.83)	0.010
Model3	1	1.19 (0.90 1.54)	0.240	1.21 (0.92 1.59)	0.176	1.42 (1.09 1.85)	0.011

The four groups of TP are expressed as Q1 (59.3–71.6), Q2 (71.7–74.0), Q3 (74.1–76.3) and Q4 (76.4–90.9). TP is presented as the median (range)

Model 1: adjustment for age and WC;

Model 2: adjustment for TC, TG, LDL-C and HDL-C + Model 1;

Model 3: adjustment for SBP, DBP, ALT, AST, BUN, TBIL, eGFR + Model 2

### Dose-response between TP and T2DM in the total population

**Fig. 2:**
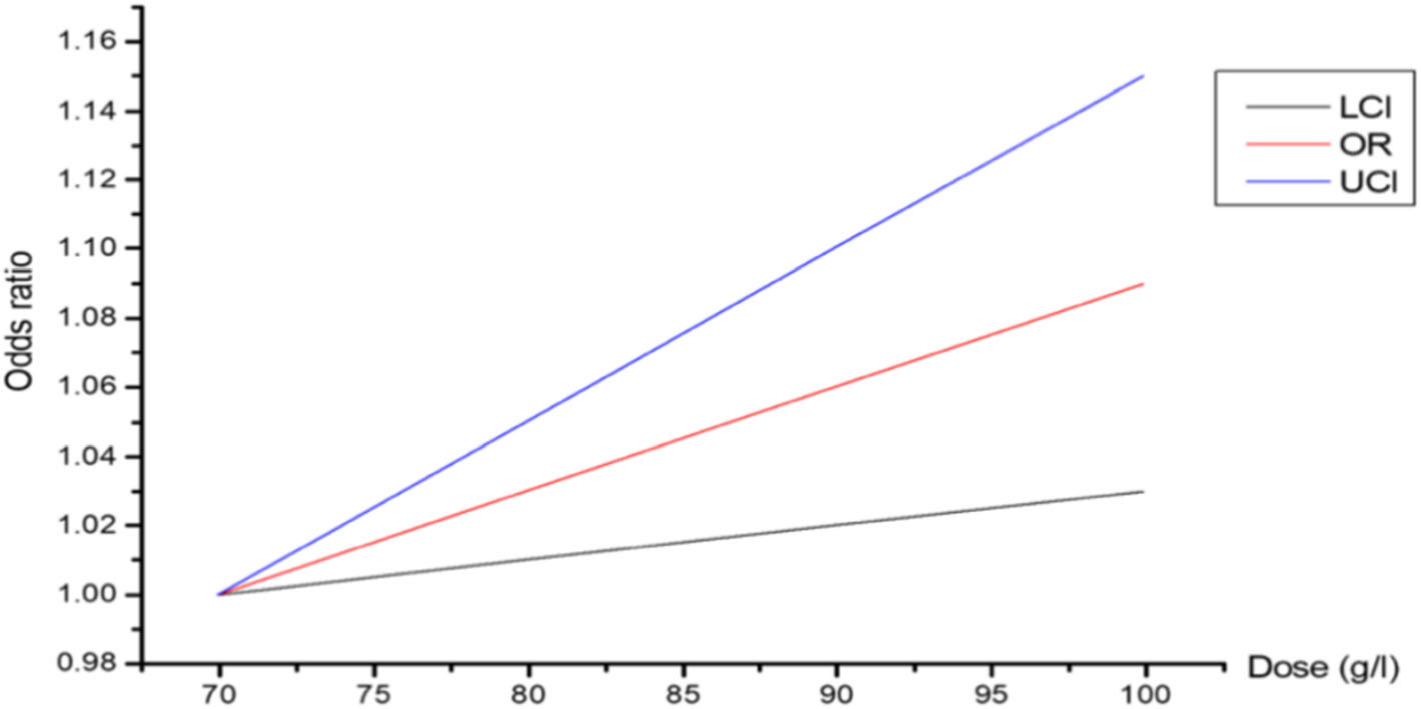
Dose-response between TP and T2DM in the total population. OR: odds ratio; UCI: Upper confidence interval; LCI:Lower confidence interval

As shown in Fig. 2, we took the dose of TP as the abscissa and the relative risk of T2DM as the ordinate. The results showed that for every 10 g/ITP increase, the risk of T2DM increased 1.03 times.

## Discussion

In this population-based study, we made systematic analyses on the risk factors of T2DM before and after menopause. TP is a major risk factor for premenopausal women with T2DM. In postmenopausal groups, the prevalence of T2DM was associated with age, BMI, blood pressure, TG, BUN, ALT and TP. Even after adjusting for potential risk factors, subjects in the highest quartile of serum TP were more likely to have T2DM compared to subjects in the lowest quartile of TP in Chinese premenopausal and postmenopausal women. To the best of our knowledge, this was the first population-based study to investigate the association between serum TP levels and T2DM in the premenopausal and postmenopausal groups.

This research concluded that the prevalence of T2DM in postmenopausal women was significantly higher than in premenopausal women. TP is the predominant risk factor for T2DM in premenopausal women. However, age, WC, SBP, DBP, TG, TP, BUN, and ALT were associated with T2DM in postmenopausal women. This study suggested that TP was an independent risk factor for T2DM in females before and after menopause. A logistic analysis revealed that as the TP concentration increased, the risk of T2DM also gradually increased. We constructed unadjusted and multivariable-adjusted models (incorporating age, WC, hypertension, ALT, BUN, and eGFR). Dose-response between TP and T2DM in the total population showed that for every 10 g/ITP increase, the risk of T2DM increased 1.03 times.

There is few study on the association of TP level with T2DM.It has been reported that the TP level in diabetic patients is higher compared to the non-diabetic population [9] because insulin can promote protein synthesis. However,the results of this study suggested that the highest quartile TP patients were more susceptible to T2DM. There are several probable mechanisms. First, TP can be used as an indicator of nutritional status [14]. Because there is increased protein synthesis and reduced protein decomposition in obese people due to high body fat and high caloric intake, the serum TP levels can increase. However, obese patients are more prone to glucose metabolism disorders. Second, high TP levels are associated with fatty liver disease [15], which is related to insulin resistance (IR). Hepatic steatosis is a characteristic response of the liver to proinflammatory cytokines such as tumour necrosis factor (TNF) [16]. Thus, fatty liver disease accompanied by elevated TP levels may reflect inflammation, which may affect the insulin signalling pathway in the liver and the whole body. In this study, high levels of TP were the only non-traditional independent risk factor for T2DM in premenopausal women. Therefore, focusing on serum TP levels is an important strategy for the prevention of T2DM in females, especially in premenopausal females.

In our study, the risk factors of T2DM in postmenopausal women included age, WC, blood pressure, dyslipidaemia, BUN, ALT, and TP. Ageing and obesity, especially concentric obesity and hypertension, are traditional risk factors according to some studies [17, 18]. However, unlike previous research, in this study, BUN was an influencing factor for T2DM in postmenopausal women. Little research has focused on the relationship between elevated BUN and T2DM. Only Xie [19] et al. built a national cohort of 1,337,452 United States veterans without diabetes to characterize the association of BUN with the risk of diabetes. Over a median follow-up of 4.93 years, there were 172,913 cases of diabetes. Spline analyses of the relationship between BUN and the risk of diabetes showed that the risk was progressively higher as BUN increased.

The mechanisms that underlie the influence of increased BUN on T2DM in postmenopausal women are still unclear. BUN is an important indicator for abnormal renal function. The kidney is an important organ in glucose homeostasis [20], and it relies on sufficient production of insulin from pancreatic beta cells to function adequately in peripheral tissues [21]. In CKD, insulin production and tissue sensitivity to insulin are impaired [22]. The underlying mechanisms of glucose homeostasis still remain unclear. The mechanisms likely involve retention of uremic metabolites, including urea and p-cresyl sulphates, modification of the gut microbiome, oxidative stress, and inflammation. Other conditions, including metabolic acidosis, ageing, and excess angiotensin II, may result in insulin resistance [23, 24]. Pham [25] et al. and de Boer [26] et al. clearly demonstrated that kidney disease leads to increased insulin resistance and decreased insulin sensitivity. Koppe [21] et al. demonstrated that insulin secretory defects associated with CKD arise from elevated circulating levels of urea that increase islet protein O-GlcNAcylation and impair glycolysis.

Another main finding of our present report showed that high ALT was an independent risk factor for T2DM in postmenopausal women. Postmenopausal women with elevated ALT levels have a higher risk of glucose metabolism disorders than those with normal ALT levels. There are few studies regarding the relationship between ALT and T2DM. Barbora Vozarova [28] et al. explained that the relationship between liver enzyme concentrations, including ALT, AST and γ-glutamyltranspeptidase (GGT), and insulin sensitivity was not associated with the severity of obesity. There are different analyses of the relationships between high ALT levels and T2DM. 1) ALT levels are not associated with insulin sensitivity or insulin secretion, decreasing in the whole body but not the liver [27, 28]. Some animal experiments suggest that isolated hepatic insulin resistance leads to a wider range of impaired glucose tolerance [28]. 2) ALT belongs to a group of gluconeogenic enzymes, which were inhibited by insulin. Therefore, elevated ALT levels may suggest an early abnormality of the insulin signalling pathway [29]. 3) As we know, liver damage can lead to increasing ALT levels, liver glycogen dysfunction, a decrease in liver glycogen reserve capacity and postprandial hyperglycaemia. Therefore, the ALT levels in postmenopausal females are crucial to prevent T2DM. However, a prospective study is necessary to determine whether reducing ALT can reduce the incidence of T2DM in postmenopausal women.

Our study differs from previous investigations. First, subjects from this cross-sectional study were Chinese district females free of CAD, peripheral arterial disease, and chronic kidney disease (CKD), and medical examinations occurred every two years. Second, a novel risk factor for T2DM, TP levels, was found in premenopausal females, whereas some non-traditional risk factors, including BUN and ALT levels, were found in postmenopausal females. Third, the study recruited 3227 individuals. To our knowledge, the present work is the first to assess the prevalence and risk of T2DM in a large Chinese population.

Our study had some limitations. First, because all of the selected respondents were women admitted to the medical examination, selection bias may have occurred. Therefore, future studies may consider expanding the sample size, improving the survey method, and having continuous follow-up of the female population before and after menopause to clarify age and hormone changes in T2DM. Second, a cross-sectional study cannot infer the causality between risk factors and T2DM in females. Therefore, more longitudinal studies are needed to investigate whether risk factors associated with T2DM in premenopausal and postmenopausal females represent a new risk pattern in the Asian population.

## Conclusions

For women, menopause is an important period because metabolic and cardiovascular disease risks increase significantly and the function of each system continues to decline due to oestrogen and progesterone level variations. In addition to traditional risk factors, such as obesity, dyslipidaemia and hypertension, we found that TP level is a predominant risk factor for T2DM in premenopausal women. However, TP, BUN and ALT were non-traditional risk factors for T2DM in postmenopausal women. Therefore, it is important to recognize and monitor the risk factors for T2DM as early as possible in females of different ages. Moreover, intervention studies are needed to investigate whether a strategy to reduce these risk factors can impact the incidence of T2DM in females.

## Data Availability

The datasets during and/or analysed during the current study available from the corresponding author on reasonable request.

## Abbreviations

ALT: Alanine aminotransferase
BMI: Body mass index
BUN: Blood urea nitrogen
mALB: Microalbuminuria
T2DM: Type 2 diabetes mellitus
TG: Triglycerides
TP: Total serum protein
WC: Waist circumference
FPG: Fasting plasma glucose
TC: Total cholesterol
HDL-C: High-density lipoprotein cholesterol
LDL-C: Low-density lipoprotein cholesterol
CREA: Creatinine UA:uric acid
AST: Aspartate transaminase
TBIL: Total bilirubin
HbA1c: Glycated haemoglobin
eGFR: Estimated glomerular filtration rate
OGTT: Oral glucose tolerance test
SD: Standard deviation
IR: Insulin resistance
TNF: Tumour necrosis factor
GGT: γ-glutamyltranspeptidase
CKD: Chronic kidney disease
